# *fs(1)A1304^1^* is a 5′ UTR deletion of the essential gene *small ovary* in* Drosophila*

**DOI:** 10.17912/micropub.biology.000246

**Published:** 2020-05-06

**Authors:** Myles Hammond, Jillian G. Gomez, Brian Oliver, Steve Kucera, Leif Benner

**Affiliations:** 1 Department of Biology, The University of Tampa, Tampa, FL, 33606; 2 Section of Developmental Genomics, Laboratory of Cellular and Developmental Biology, National Institute of Diabetes and Digestive and Kidney Diseases, National Institutes of Health, Bethesda, MD, 20892; 3 Department of Biology, Johns Hopkins University, Baltimore, MD, 21218

**Figure 1:**
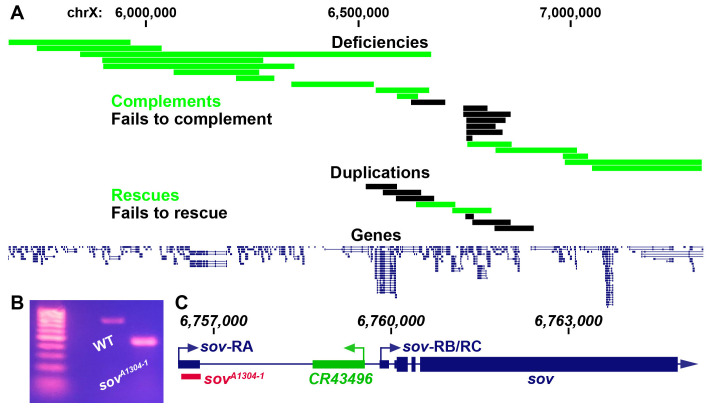
A) Complementation mapping of *fs(1)A1304^1^*. Boxes represent either the portion of the chromosome deleted or duplicated. For deficiencies, green indicates complementing deletions and black indicates non-complementing deletions. For duplications, green indicates rescuing fragments while black indicates non-rescuing fragments. Numbers indicate genomic coordinates in bases along the X chromosome. B) Genomic PCR of wildtype (WT) and *sov^A1304-1^* flies. Primers were designed to amplify genomic DNA encoding the 5′ UTR region of the *sov*-RA transcript. C) Cartoon of the *sov* locus. Dark blue represents the *sov* gene region with the left arrow representing the *sov*-RA transcriptional start site and right arrow representing the *sov*-RB/RC transcriptional start site. Green represents the *CR43496* gene region with the arrow representing the transcriptional start site. Red box represents the deleted segment in *sov^A1304-1^* flies. Small rectangles represent untranslated regions while large boxes represent translated regions. Numbers indicate genomic coordinates in bases along the X chromosome.

## Description

X-linked female sterile screens in *Drosophila* have led to a tremendous increase in our understanding of the genetic control of oogenesis (Gans *et al.* 1975; Mohler 1977; Komitopoulou *et al.* 1983). However, many of the loci in these screens have not been mapped to a single gene and therefore remain a rich resource for further elucidating the genetic control of female fertility. *fs(1)A1304^1^* is one such allele that is germline dependent and results in a degenerative ovary phenotype (Gans *et al.* 1975; Khipple and King 1976; Mulligan 1981; Wieschaus *et al.* 1981; Mulligan and Rasch 1985; Lamnissou and Gelti-Douka 1985). We were interested in determining the mutation that leads to sterility in *fs(1)A1304^1^* females. Previous recombination mapping had placed *fs(1)A1304^1^* at 19±2 cM on the X chromosome (Gans, Audit, and Masson 1975; Khipple and King 1976). We confirmed the previous mapping interval by meiotically mapping *fs(1)A1304^1^* to the right of *crossveinless* (12 cM) and to the left of *singed* (22 cM). We began complementation tests for female sterility with known deficiencies tiling the *crossveinless* and *singed* region and placed the lesion within a roughly 235 kb region (Figure 1A, non-complementing *Df(1)BSC276*, *BSC285*, *BSC286*, *BSC297*, *BSC351*, *BSC535*, and *sov*) (Parks *et al.* 2004; Cook *et al.* 2012). Two duplications within this narrow region rescued *fs(1)A1304^1^* sterility and thus further narrowed down the possible location of the causal mutation (Figure 1A, *Dp(1;3)DC486* and *Dp(1;3)DC026*) (Venken *et al.* 2010). The mapping results were somewhat ambiguous within this narrow region (discussed below). However, the smallest non-complementing deficiency, *Df(1)sov*, contains only the protein coding gene *small ovary* (*sov*) and non-coding RNA gene *CR43496*. We therefore decided to complementation test *fs(1)A1304^1^* with known alleles of *sov*. Flies homozygous for hypomorphic alleles of *sov* show a similar female sterility phenotype to flies bearing *fs(1)A1304^1^* while amorphic *sov* alleles are embryonic lethal (Wayne *et al.* 1995; Jankovics *et al.* 2018; Benner *et al.* 2019). We found that amorphic alleles *sov^EA42^* and *sov^ML150^* failed to complement *fs(1)A1304^1^* female sterility while the hypomorphic *sov^2^* complemented *fs(1)A1304^1^* sterility. Collectively this indicates that *fs(1)A1304^1^* is a *sov* allele (*sov^A1304-1^*).

To determine the molecular lesion, we performed paired-end DNA sequencing on *sov^A1304-1^* females. The *sov* locus contains three annotated transcripts; *sov*-RA has an annotated upstream transcriptional start site while *sov*-RB/RC are annotated to use a downstream transcriptional start site (Thurmond *et al.* 2019). Our sequencing data suggested that *sov^A1304-1^* flies contained a deletion within the *sov* gene region that would delete a majority of the *sov*-RA 5′ UTR. Genomic PCR of this potential deletion confirmed the presence of a deletion in *sov^A1304-1^* flies (Figure 1B). Sanger sequencing of the *sov^A1304-1 ^*genomic PCR product showed that there was a 324 nucleotide deletion (chrX:6,756,385-6,756,709) and a 10 nucleotide insertion (TCAACCTTCG) in the *sov*-RA 5′ UTR and would therefore remove most of the annotated 5′ UTR and donor splice site (Figure 1C).

We are unsure why a duplication (*Dp(1;3)DC026*) and a deficiency (*Df(1)BSC535*) to the left of the *sov* region rescued and failed to complement *sov^A1304-1^*, respectively. We also found that the small duplication of just *sov* and *CR43496* (*Dp(1;3)sov^tCH322-191E24^*) failed to rescue. We were not able to find any deleterious mutations or structural variants in our sequencing data to the left of *sov* that might indicate the presence of a second-site suppressor or long-range genomic interactions with the *sov* locus that are necessary for its proper expression. It is interesting that *sov^A1304-1^* had not been previously mapped to *sov* since the Mohler and Gans X-linked female sterile collections had been previously complementation tested *inter se* (Perrimon *et al.* 1986). We found that one of the original Mohler alleles, *sov^2^*, complemented *sov^A1304-1^*sterility and is thus possible that the other two Mohler alleles, *sov^1^* and *sov^3^*, behaved similarly, providing an explanation as to why *sov^A1304-1^* was not previously recognized as belonging to the *sov* locus. It would be interesting to determine if the 5′ UTR deletion of the *sov*-RA transcript found in *sov^A1304-1^* flies affects *sov* activity in other tissues of the body other than the ovary. There is no indication that *sov*-RA, or *sov*-RB/RC, is differentially expressed in the ovary or other adult tissues (Benner *et al.* 2019). Pole cell transplantation studies of *sov^A1304-1^* indicated that defects are germline dependent (Wieschaus *et al.* 1981; Lamnissou and Gelti-Douka 1985), however, *sov* is an essential gene that has been shown to dominantly suppress position-effect variegation in tissues such as the eye (Jankovics *et al.* 2018; Benner *et al.* 2019). It is possible that the deletion solely affects *sov*-RA and that the *Drosophila* ovary is more sensitive to loss of *sov*-RA, or *sov* transcripts in general, in comparison to other tissues since *sov^A1304-1^* females are viable but sterile. However, we have not directly measured the deletions effects on *sov*-RB/RC transcript levels, which might also be perturbed. The nature of the *sov^A1304-1^* deletion therefore provides a unique mechanism to further elucidate the function of Sov at potentially both the transcript and regulatory level in *Drosophila*.

## Methods

Flies were cultured on ‘Fly Food A’ (LabExpress, Ann Arbor, MI) under standard laboratory conditions at 25°C. Genomic DNA was extracted from 30 homozygous *fs(1)A1304^1^* flies with a Qiagen DNeasy Blood and Tissue Kit (Hilden, Germany) according to the manufacturers insect protocol. DNA-sequencing libraries were made with Illumina Nextera DNA Library Prep Kit (San Diego, CA). 50 nucleotide paired-end sequencing was performed (Illumina HiSeq 2500, CASAVA base calling). Sequencing reads were mapped with Hisat2 to the FlyBase r6.25 genome and are available at the SRA (SRP238927) (Kim *et al.* 2015; Thurmond *et al.* 2019). Variant calling was completed with mpileup and bcftools from SAMtools within the X chromosome region 6625450-6860753 (Li *et al.* 2009; Li 2011) followed with variant annotation software snpEFF (Cingolani *et al.* 2012). For structural variant calling, we used BreakDancer software (Chen *et al.* 2009). Sanger sequencing was completed by Genewiz (Plainfield, NJ).

## Reagents

Deficiencies and duplications in order as they appear in Figure 1 (top to bottom).

Deficiencies:

*Df(1)ED6802* = BDSC 8949 (or FBst0008949)

*Df(1)BSC654* = BDSC 26506 (or FBst0026506)

*Df(1)dx81* = BDSC 5281 (or FBst0005281)

*Df(1)ED418* = BDSC 8032 (or FBst0008032)

*Df(1)ED6829* = BDSC 8947 (or FBst0008947)

*Df(1)Exel6238* = BDSC 7712 (or FBst0007712)

*Df(1)BSC640* = BDSC 25730 (or FBst0025730)

*Df(1)Exel6239* = BDSC 7713 (or FBst0007713)

*Df(1)Exel6240* = BDSC 7714 (or FBst0007714)

*Df(1)e02477-d06059* = BDSC 39617 (or FBst0039617)

*Df(1)BSC535* = BDSC 25063 (or FBst0025063)

*Df(1)BSC285* = BDSC 23670 (or FBst0023670)

*Df(1)BSC351* = BDSC 24375 (or FBst0024375)

*Df(1)BSC297* = BDSC 23681 (or FBst0023681)

*Df(1)BSC286* = BDSC 23671 (or FBst0023671)

*Df(1)BSC276* = BDSC 23661 (or FBst0023661)

*Df(1)sov* = Benner *et al.*, 2019

*Df(1)ED6878* = BDSC 9625 (or FBst0009625)

*Df(1)BSC882* = BDSC 30587 (or FBst0030587)

*Df(1)BSC867* = BDSC 29990 (or FBst0029990)

*Df(1)Sxl-bt* = BDSC 3196 (or FBst0003196)

*Df(1)Sxl^fP7B0^* = BDSC 58489 (or FBst0058489)

Duplications:

*Dp(1;3)DC158* = BDSC 30296 (or FBst0030296)

*Dp(1;3)DC159* = BDSC 32268 (or FBst0032268)

*Dp(1;3)DC160* = BDSC 30297 (or FBst0030297

*Dp(1;3)DC026* = BDSC 30226 (or FBst0030226)

*Dp(1;3)DC486* = BDSC 32306 (or FBst0032306)

*Dp(1;3)sov^tCH322-191E24^* = Venken *et al.*, 2010 (or FBal0243261)

*Dp(1;3)DC163* = BDSC 32269 (or FBst0032269)

*Dp(1;3)DC164* = BDSC 32270 (or FBst0032270)

Alleles:

*fs(1)A1304^1^* (*sov^A1304-1^*) = BDSC 4314 (or FBst0004314)

*sov^2^* = BDSC 4611 (or FBst0004611)

*sov^EA42^* (synonymous with *l(1)6Dc^3^*) = FBal0007068

*sov^ML150^* = BDSC 4591 (or FBst0004591)

Primer *fs(1)A1304^1^* Forward = TGACCATGTTGCATCTAAGCCA

Primer *fs(1)A1304^1^* Reverse = AGTAGAGCTCGCAATACGCC
